# Implication of Crystal Water Molecules in Inhibitor Binding at ALR2 Active Site

**DOI:** 10.1155/2012/541594

**Published:** 2012-05-08

**Authors:** Hymavati, Vivek Kumar, M. Elizabeth Sobhia

**Affiliations:** Department of Pharmacoinformatics, National Institute of Pharmaceutical Education and Research (NIPER), Sector 67, Punjab 160 062, S.A.S Nagar, India

## Abstract

Water molecules play a crucial role in mediating the interaction between a ligand and a macromolecule. The solvent environment around such biomolecule controls their structure and plays important role in protein-ligand interactions. An understanding of the nature and role of these water molecules in the active site of a protein could greatly increase the efficiency of rational drug design approaches. We have performed the comparative crystal structure analysis of aldose reductase to understand the role of crystal water in protein-ligand interaction. Molecular dynamics simulation has shown the versatile nature of water molecules in bridge H bonding during interaction. Occupancy and life time of water molecules depend on the type of cocrystallized ligand present in the structure. The information may be useful in rational approach to customize the ligand, and thereby longer occupancy and life time for bridge H-bonding.

## 1. Introduction

Diabetes is a debilitating disease leading to severe complications and a shortened life expectancy. Diabetes-specific microvascular disease leads to blindness, renal failure and nerve damage, and diabetes-accelerated atherosclerosis which increase risk of myocardial infarction, stroke, and limb amputation [1]. The insulin therapy for tissues that do not require insulin does not prevent complications such as neuropathy, retinopathy, nephropathy, and cataracts [2]. Large prospective clinical studies show a strong relationship between glycaemia and diabetic microvascular complications in both type 1 and type 2 diabetes [3]. Hyperglycaemia and insulin resistance are suggested to play important roles in the pathogenesis of macrovascular complications [4].

**Figure 1:**
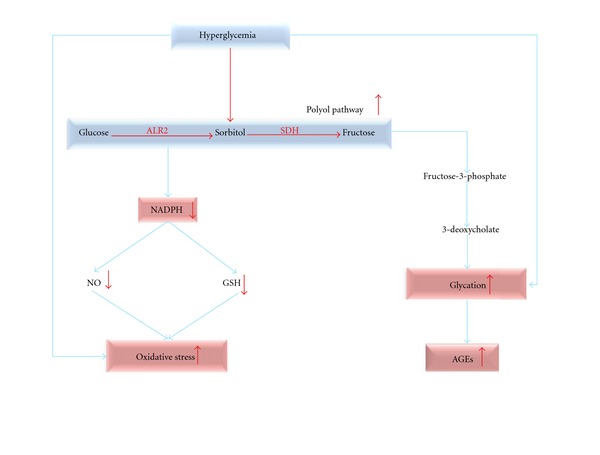
Hyperglycemia pathway.

Aldose reductase 2 (ALR2, alditol: NAD(P) 1-oxidoreductase, EC 1.1.1.21) is the first enzyme in the polyol pathway that catalyses the NADPH-dependent reduction of D glucose to D sorbitol [5]. It is a cytosolic, monomeric oxidoreductase that catalyses the NADPH-dependent reduction of wide variety of carbonyl compounds including glucose [6]. Under diabetic hyperglycaemia, excess glucose is metabolized by the polyol pathway (Figure 1). This pathway comprised of two enzymes, namely, ALR2 which reduces excess D-glucose into D-sorbitol, and sorbitol dehydrogenase which converts sorbitol to fructose. Diabetic complications have been linked to excessive accumulation of sorbitol, and targeting the polyol pathway by inhibiting ALR2 offers an option for treatment [7].

In normal physiological conditions, ALR2 is involved in osmoregulation while under hyperglycaemic conditions it contributes to the onset and development of severe complications in diabetes [8]. The elevated glucose level enhances the activity of ALR2 by directly increasing the glucose flux through this pathway and indirectly by forming reactive oxygen species (ROS), which activates ALR2 [9]. The increased activity of ALR2 results in decreased NADPH/NADP^+^ ratio, and it affects other NADPH-dependent enzymes, such as nitric oxide (NO) synthase and glutathione reductase [10]. The reduction of NO level leads to decreases nerve conduction and microvascular derangement. The retarded activity of the antioxidative enzyme glutathione reductase causes oxidative stress under diabetic conditions [11]. Inhibiting ALR2 would provide a way of avoiding diabetic complications, and, therefore, identifying inhibitors is an important pharmaceutical goal.

Epalrestat is the only inhibitor of aldose reductase that is successfully marketed in Japan for treatment of diabetic neuropathy. Many promising compounds from different *in vitro *and *in vivo* studies have failed to proceed beyond clinical trials. The lack of efficacy or adverse effects as a result of less-specific inhibitor, and a likely inhibition of the related aldehyde reductases are the major bottlenecks in the development of ALR2 inhibitors. The knowledge of catalytic mechanism and available structures information of current inhibitors coupled with ALR2 can help in accelerating the discovery or designing of specific inhibitors. A limited number of currently available drugs for the treatment of diabetic complications only suggests the importance of research for new ALRIs [12].

**Figure 2:**
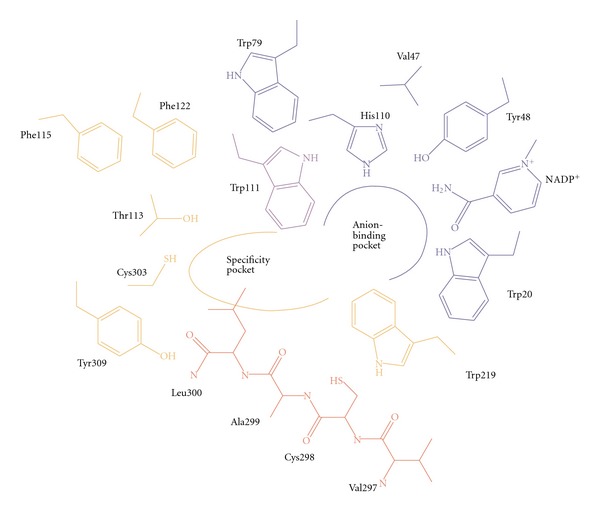
Schematic drawing of ALR2 binding pocket.

ALR2 is a monomer (35.8 kDa), encoded by a single gene located on chromosome region 7q35.ALR2, made up of a single polypeptide chain with 315 residues. The crystal is a single domain structure folded into an eight-stranded parallel *α*/*β* motif. The substrate-binding site is located in a cleft at the carboxy terminal end of the *β*-barrel and involves loop residues. The large and highly hydrophobic active site is located at the carboxy terminus of (*β*/*α*)_8_ barrel. To date, there are 95 crystals of ALR2 deposited in Protein Data Bank [13]. The ligand-binding site is a large, deep, elliptical pocket with the nicotinamide ring of NADPH cofactor lying at the base. The enzyme has anion-binding pocket and specificity pocket (Figure 2). The anion-binding pocket has Tyr 48, His110, Trp111, Trp 20, Phe122, and Trp 219 while the specificity pocket is lined by residues Trp111, Thr113, Phe122, Ala 299, and Leu 300.

We present here molecular dynamics simulation study on the crystal structure of ALR2 enzyme. Moreover, the goal of our study is to evaluate the importance of the crystal water molecule in the active site of the ALR2. These water molecules mediate the hydrogen bond and help ALRIs to bind at the active site.

## 2. Materials and Methods

### 2.1. Comparative ALR Crystal Structure Analysis

The 87 crystal structures of human ALR were obtained from the Protein Data Bank (PDB) [13]. The crystals were aligned by “Align by homology” program in Biopolymer module in Sybyl7.1, taking the apoenzyme (PDB id: 1ADS) as the reference structure [14]. The crystals were analyzed for the presence of ordered/conserved water molecules in the active site using PyMol [15].

### 2.2. Preparation of Input Files for MD Simulation

Among all available crystal structures of human ALR2, five crystal structures having different ligand (PDB id: 1T40, 1Z89, 2FZ8, 3H4G, and 3G5E) were selected to understand the role of water molecules in the binding of ligands with human ALR2 [13]. Coordinates of crystal structures were taken from the PDB. Missing residues were added, and mutated residues were corrected to wild-type residues using Schrodinger *maestro* package [16]. Missing hydrogen atoms of ligands and cofactors were added using Schrodinger *maestro *package. AM1-BCC charges were used for the ligands as well as cofactor NADPH, calculated using Antechamber program. Parameters for the ligands were generated using the general amber force field (GAFF) and Antechamber module of AMBER while ff99SB force field was used for protein. Missing hydrogen atoms of the protein structures were added using LeaP module of AMBER. Amino acids were kept in their default ionization states. Crystal water molecules were kept as such while preparing the system. Complex was solvated using orthorhombic TIP3P water box, distance between the edge of the box, and periphery of the protein is 8 Angstrom [17]. Solvated systems were neutralized by adding required number of counter ions. A two-stage approach was adopted for minimizing the protein. In the first stage, the protein, ligand, and cofactor were kept fixed with weak restraint (10 kcal/mol-Å^2^), and only solvent was allowed to minimized. Then, in the second stage, the entire system was minimized. Initially, minimization of 1000 steps of steepest descent was carried out and this was followed by another 500 steps of conjugate gradient minimization. Thereafter, minimizing solvent, the entire system consisting water, protein, and ligand cofactor was minimized for 2500 steps without restraint.

After minimization process, the system was heated from 0 to 300 K. In order to avoid any instability during the MD production run, an initial MD run were carried out in NPT ensemble (pressure = 1 bar, temperature = 300 Kel) to equilibrate the system for 20 ps with weak restraints on the solute. Production run were carried out for 5 ns time, using NVT ensemble and Langevin dynamics used to control the temperature through a collision frequency of 1.0 ps^−1^ [18]. Step size was kept 2 fs for the throughout simulations. A SHAKE algorithm was applied to constrain bonds involving hydrogen atoms [19]. The van der Waals cutoff was kept 8 angstrom and long range electrostatic interactions were treated using the Particle Mesh Ewald (PME) method. Co-ordinates were saved after each 1000 step, which were finally used for analysis.

All simulations were carried out using SANDER program of AMBER10 package. Analysis was performed using VMD and Ptraj module of Amber tools [20]. Additionally, Chimera and Pymol were used for molecular visualization [21].

### 2.3. MM-PB (GB) SA Calculations

MMPBSA calculations were performed on 5 complexes: 1T4O, 1Z89, 2FZ8, 3H4G and 3G5E. The interaction energy and salvation-free energy for the complex, receptor, and ligand were calculated with the help of the snapshots extracted at each 10 ps from the 1.5 ns to 5 ns run. The average of the results was calculated to get an estimate of the binding free energy. The binding energy calculation was carried out with MM_PBSA and MM_GBSA method for the sake of comparison. Additionally, binding energies were also estimated by MM-PBSA program of Prime module of Schrodinger *maestro *package.

## 3. Results and Discussion

### 3.1. Comparative Crystal Structure Analysis of ALR2 Complexes

All the 87 crystal structures of human ALR2 were aligned by “Align by homology” program in Biopolymer module in Sybyl7.1, taking the apoenzyme, 1ADS as a reference structure. This module aligns proteins on the basis of their sequence similarity. The tailor variables control the gap penalty, the number of jumbles, and similarity matrix used. The sequences were compared by superimposing the C*α*, backbone, side chain, and all atoms. The crystal structures having a RMSD value greater than 0.5 Å were rejected. The selected 28 crystal structures were analyzed using Accelrys Discovery Studio 2.0 [22] and Pymol to visualize and identify atoms participating in the ligand binding including the water molecules in the active site. After analyzing the atoms, 23 crystal structures were found to have water molecules in the active site which showed hydrogen bond interactions.

The criterion in selecting the hydrogen bonds was the bond length that should not be greater than $3\;\overset{´}{\text{Å}}$. The active site was observed to have more number of bridging water molecules within the anionic pocket of NADPH rather than the ligand (Table 1). We assumed that the phosphate groups present in the NADPH are responsible for it. The ligands were found to interact with at least one water molecule in the active site. The water molecules are said to be bridging if they simultaneously make hydrogen bonds with both ligand and amino acid residues.

### 3.2. Analysis of Ligand-Binding Site

**Figure 3:**
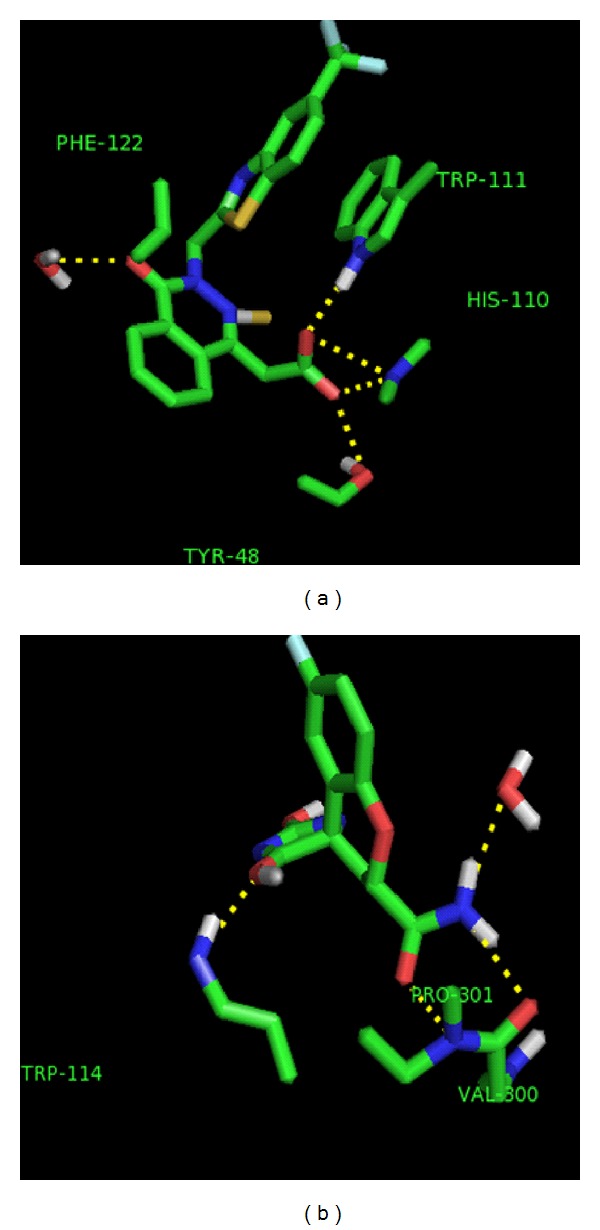
Water molecule interacting with ligand in crystal structure (a) 2FZ8 and (b) 3H4G. The ligand and the amino acid residues in the active site are represented in stick. The yellow broken line represents the hydrogen bond between the ligand and the active site residues.

The crystal structures, 1T40, 1Z89, 2FZ8, 3H4G, and 3G5E, were selected for further studies. The active sites were analyzed around 5 Å region of ligand to identify the number of water molecules and hydrogen bond interactions with corresponding ligand. It was noted that most of the structures have at least one water molecule in the active site which shows hydrogen bond interactions with the ligand. In Figure 3, the ligands of 2FZ8 and 3H4G make a hydrogen bond with the water molecule at the active site. All the ligands, except in 1Z89, displaced the 9 ordered water molecules in the active site of the apoenzyme, whereas the ligand in 1Z89 displaced 6 water molecules. These differences can be attributed to the size and conformation of the ligand in the active site. The 5 complexes, upon superimposing on the apoenzyme, showed their ligands, displacing the 6 ordered water molecules in the active site of the apoenzyme. The size of the ligand from 3H4G is exactly equal to the size occupied by the 6 ordered water molecules in the apoenzyme; whereas, the other ligands have an extended conformation in both the anionic and specificity site.

### 3.3. Binding Free Energy Calculations

Explicit solvent MD simulations were carried out for all complexes under study. The absolute binding free energy of the complex formation was estimated from energetic and entropic contributions, and calculated for snapshots extracted from the trajectories. The snapshots of the unbound proteins and complexes were taken from molecular dynamics (MD) and were further processed for 2 ns using periodic boundary conditions. The snapshots of the unbound molecules were extracted only from the trajectory of the solvated complex. A total of 1000 snapshots were extracted from the 2 ns trajectory, with 200 snapshots for each of the solvated complex.

Solvation free energies were computed as the sum of polar and nonpolar contribution, using a continuum representation of the solvent. The polar contribution was calculated by solving the Poisson equation. The nonpolar contribution to the solvation free energy due to cavity formation and van der Waals interactions, between the solute and the solvent, was estimated by a solvent-accessible surface area. The total binding energy was negative in all the complexes, and it signifies a favorable protein-ligand complexes. The results for the binding free energy after performing simulation for 2 ns are shown in Table 2. Although the result shows zopolrestat, the second potent ligand (2FZ8) has the highest binding affinity, while sulfonyl pyridazinone (1Z89) has the lowest binding affinity. The inhibitors have maximum potency difference of 2.8 between most and least potent ligand at scale of pIC_50_. It indicates coinciding nature of biological activity which merely expected to be demarcated by correlation with predicted binding free energy due to lack of wide range. 

The electrostatic contribution to solvation free energy in the 5 complexes is shown in Table 3. The Δ*G*_ele_ and Δ*G*_vdW_ are electrostatic (ELE) and van der Waals (VDW) contribution, calculated by the MM force field respectively. The Δ*G*_ele-int_ is the internal energy (INT) arising from bond, angle, and dihedral terms in the MM force field. The sum of ELE, VDW, and INT is known as total gas phase energy. The Δ*G*_non-polar_ is the non-polar contribution to the solvation free energy calculated by an empirical model while Δ*G*_ele-MM_, is sum of the electrostatic solvation free energy and MM electrostatic energy. 1Z89 makes the highest contribution to the solvation free energy while 3G5E makes the lowest. The nonpolar contributions to the solvation are similar in all the five complexes. Electrostatic component of the solvation free energy was calculated by GB model. Polar contributions to the solvation free energy were computed by applying linear PB model and an extension to the GB model. It disfavors binding of the protein ligand complexes in ALR2. PB and GB calculations give very similar results in that respect. 

Moreover, the Δ*G*_bind_ calculated for the ALR2 crystal structure with and without crystal molecules was found reasonable. The binding energy of the ALR2 complexes without crystal water was observed low by 3–7 kcal/mol as compared to complexes with water. It clearly indicated that crystal water molecules helped in making strong and stable protein-ligand complexes (Table 3). 

### 3.4. Molecular Dynamics Simulation with Crystallized Water Molecules

**Figure 4:**
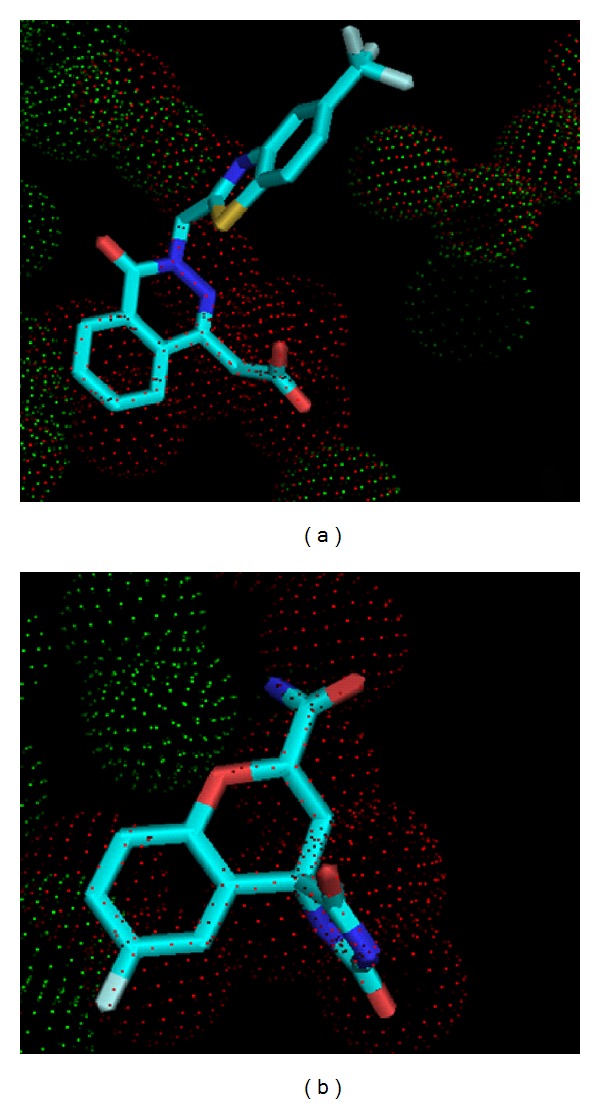
Water molecules present in the apoenzyme (red dot) displaced by the ligand in crystal structure (green dot) (a) 2FZ8 and (b) 3H4G. The ligand in the active site is represented in stick.

Explicit simulations were carried out for the crystal structures—1Z89, 2FZ8 and 3H4G (Figure 4). The crystallized water molecules were retained in this simulation. The notion of crystal waters is tricky in MD simulation since water is highly mobile and therefore, they exchange rapidly. Even at tight interfaces, such as a protein-nucleic acid interface, the lifetimes of bound water are on the nanosecond time scale. Hence, the waters in the solvation shell around the ligand were calculated after processing of the trajectories with ptraj module of AMBER. The output for this functionality contains number of waters in the first shell and second shell. The first solvation shell represents a distance of 3.4 Å from the solute, that is, ligand of interest while the second solvation shell represents a distance of 5 Å from the center of mass of the ligand. The analysis is presented separately for each complex. 

#### 3.4.1. Analysis for the Complex 1Z89

**Figure 5:**
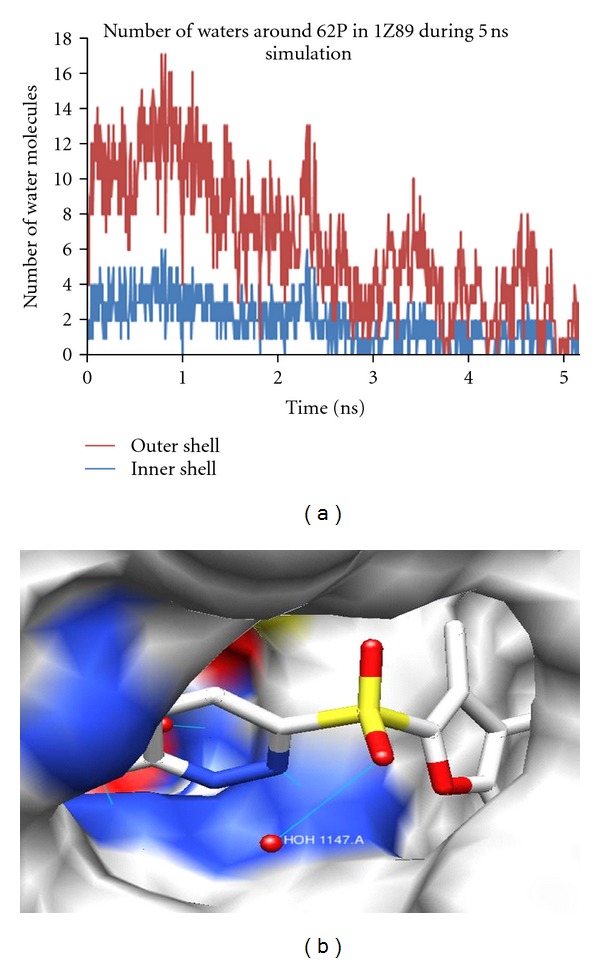
(a) Number of water molecules fluctuating in the solvation shell of 62P during 5 ns simulation and (b) ligand 62P interacting with HOH1147.

1Z89 is a human ALR2, coupled with novel sulfonyl-pyridazinone (62P), having a resolution of 0.95 Å. The pyridazinone group of the inhibitor occupies the catalytic site, whereas the chlorobenzofuran moiety penetrates the open specificity pocket. The pyridazinone exhibits a binding affinity similar to that of tolrestat and sorbinil, showing slightly reduced affinity compared to IDD594. The 62P displaces the 6 ordered water molecules which are present in the apoenzyme 1ADS, whereas other ligands replaced 9 water molecules in the apoenzyme. From the analysis of the crystal, it was observed that O18 of the ligand made hydrogen bond interactions with HOH1147. The other hydrogen bond interactions were observed for O7 with OH of Tyr48, and NE2 of His110 and N4 with NE1 of Trp111. As the simulation progressed the positions of water molecules fluctuated as indicated by graph from Figure 5(a). The graph shows the movement of water molecules within the first and second solvation shell throughout the simulation. The water molecule is stable for the location of the oxygen atom, but it tumbles freely with the hydrogen atoms jumping between several positions of the hydrogen bond network. The plot for the number of water molecules fluctuating throughout the simulation during the explicit 5 ns is shown in Figure 5(a). 

The number of water molecules in the first solvation shell was found to be 1 on an average throughout the 5 ns simulation. However, in the second solvation shell, there were 3 water molecules on an average. To understand the water association, visual analysis of the areas of high occupancy or search, a specific water interaction is useful (Table 4). The occupancy is defined as the percentage over the whole trajectory in which both the distance and the angle criteria are satisfied. 

A grid of 100 angstroms was generated around the solute. The structure was fitted to a common reference frame, by RMS fit to the first frame, to all the solute molecules in the protein. If the molecule is tumbling in space that fixed water will move with the molecule but the grid does not, it is fixed at the first frames location. If the water in question is in the same position with respect to the biomolecule, then the water molecules will move with the side chain. Since the grid did not move, the density can be smeared across multiple grid elements. This will give an idea of the probable involvement of water molecules in the active site of the protein as the simulation progresses. 

The hydration site was constructed from the water density by using the coordinate system local to each hydration site. The water structure is broken down into hydration sites constructed from water density from the protein surface. This gives the structural and dynamic properties of the water molecules from the explicit simulations. Properties studied include site occupancy, number of neighboring waters and hydrogen bonds. The occupancy of water around the ligand sulfonyl pyridazinone is analyzed by Chimera. From the density map, it was observed that the density is concentrated around O18 and O7 of the ligand, which in the native crystal was making a hydrogen bond with the water molecule. 

It is shown in the surface representation of the protein that ligand is making a hydrogen bond with a water molecule, HOH 1147 (Figure 5(b)). In this simulation, the crystal waters were removed, but the water density around the ligand shows the positions of the water molecule being retained, that is, they were occupied by the water molecules which were added explicitly. The density was at the same position where the ligand was making a bond with the water molecule, and the same pattern was observed in the remaining complexes also. This shows that positions of the water molecules are conserved in all the complexes belonging to this family. 

#### 3.4.2. Analysis for the Complex 2FZ8

The bound zopolrestat occupies almost the entire active site pocket at the C-terminal end of the *β* barrel. The inhibitor makes an unusually large number of contacts with the active site. It made a total of 132 contacts within ≤4 Å, 110 with 15 residues, 13 with the nicotinamide moiety of the coenzyme, and 9 with four ordered water molecules coenzyme. This contributes favorable entropic effect to the tight binding of the inhibitor. From our analysis, we found out that the ligand displaces 9 water molecules present in the apoenzyme. The O1 of the ligand makes a hydrogen bond with HOH1082. The other water molecules were also present in the active site, but they did not make any interactions with the ligand. The other hydrogen bond interactions were made between O3 of the ligand with OH of Tyr 48, and NE2 of His110; O2 of ZST with NE1 of Trp111, and N3 with N of Leu300. 

**Figure 6:**
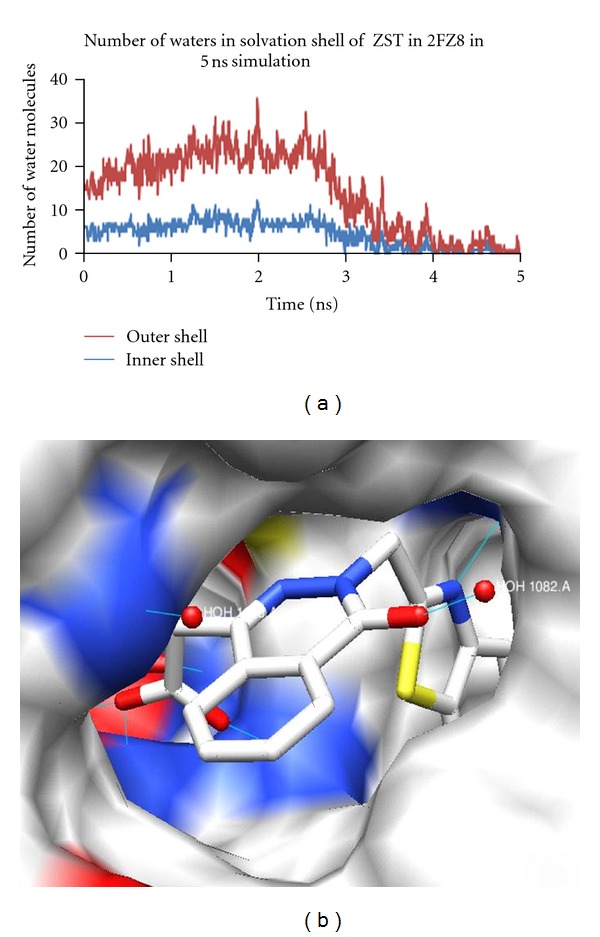
(a) Number of water molecules fluctuating in the solvation shell of ZST during 5 ns simulation, and (b) ligand ZST interacting with HOH1082.

The neighboring water molecules in the active site were studied. The number of waters in the first solvation shell, around the ligand in 2FZ8, was 6 throughout the simulation, whereas, in the second solvation shell it was 13 on an average. The number of water molecules in the first and second solvation shell, is plotted as the number of water molecules against the number of frames in the graph (Figure 6). 

The occupancy of water oxygen atom with the ligand throughout 5 ns simulation per frame is shown in Table 5. The highest occupancy was approximately 53% for 2FZ8 in different frames during the simulation, and it gradually reduced to 15% as the simulation progressed. 

#### 3.4.3. Analysis for the Complex 3H4G

3H4G is an ALR crystal structure with Fidarestat (FID), which was shown to have implications in inhibitor binding and selectivity. The ligand displaces 9 water molecules in the active site of the apoenzyme. A bridging water molecule HOH2275 is present between the ligand and the protein, making hydrogen bond interactions with N21, O10 of FID, and HOH 2015. The O6I of FID makes a hydrogen bond with NE1 of Trp114. Also, O10 of FID makes a hydrogen bond with HOH 2196, and O3 of the ligand with OH of Tyr 50. 

**Figure 7:**
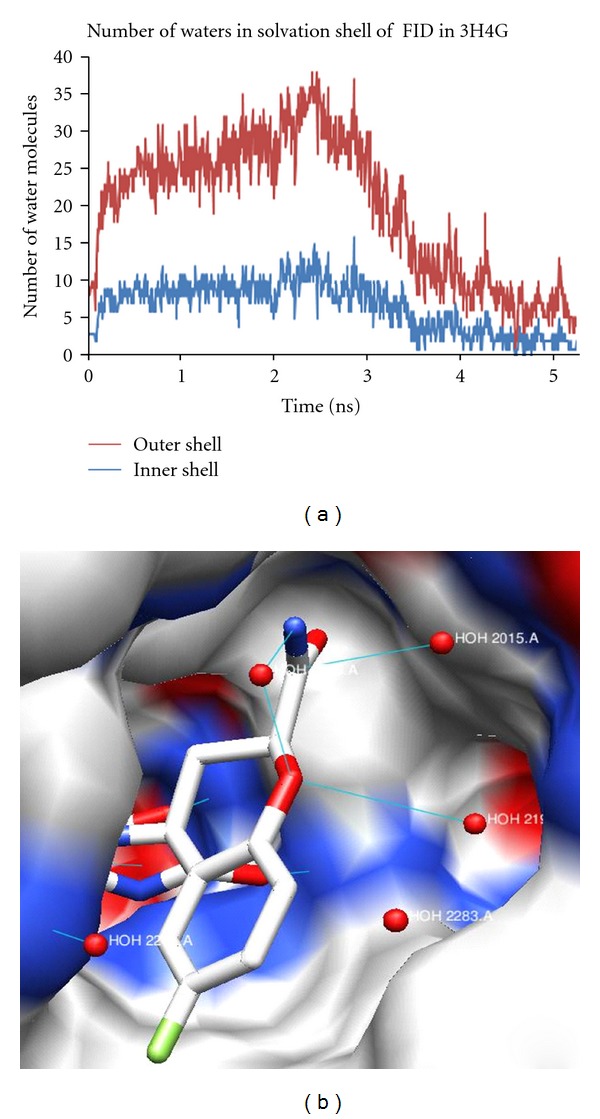
(a) Number of water molecules fluctuating in the solvation shell of FID during 5 ns simulation and, (b) ligand FID interacts with HOH2275, HOH2015 and HOH2196.

The extent of solvent exposure for each site is measured by the average number of neighboring waters within the cut-off distance 3.5 Å and 5 Å, of all waters that exist in the ligand active site, as shown in the graph (Figure 7). The number of water molecules in the first solvation shell around the ligand in 3H4G was 3 throughout the simulation, whereas it was 6 at an average in the second solvation shell.  Figure 6(b) shows a picture of the protein surface, and the ligand in stick representation, interacting with water molecules displayed as red spheres. Here, the ligand is interacting with more than one water molecule in the active site. 

Table 6 shows the occupancy of water oxygen atoms with the ligand throughout the simulation for different frames. The occupancy and the hydrogen bond distances were monitored throughout the simulation. Initially, the highest occupancy was 98%, and it got reduced to 89% as the simulation progressed. The occupancy of water in this protein was the highest when compared to other proteins in this study, and this also confirmed the sharp hydration sites. 

**Figure 8:**
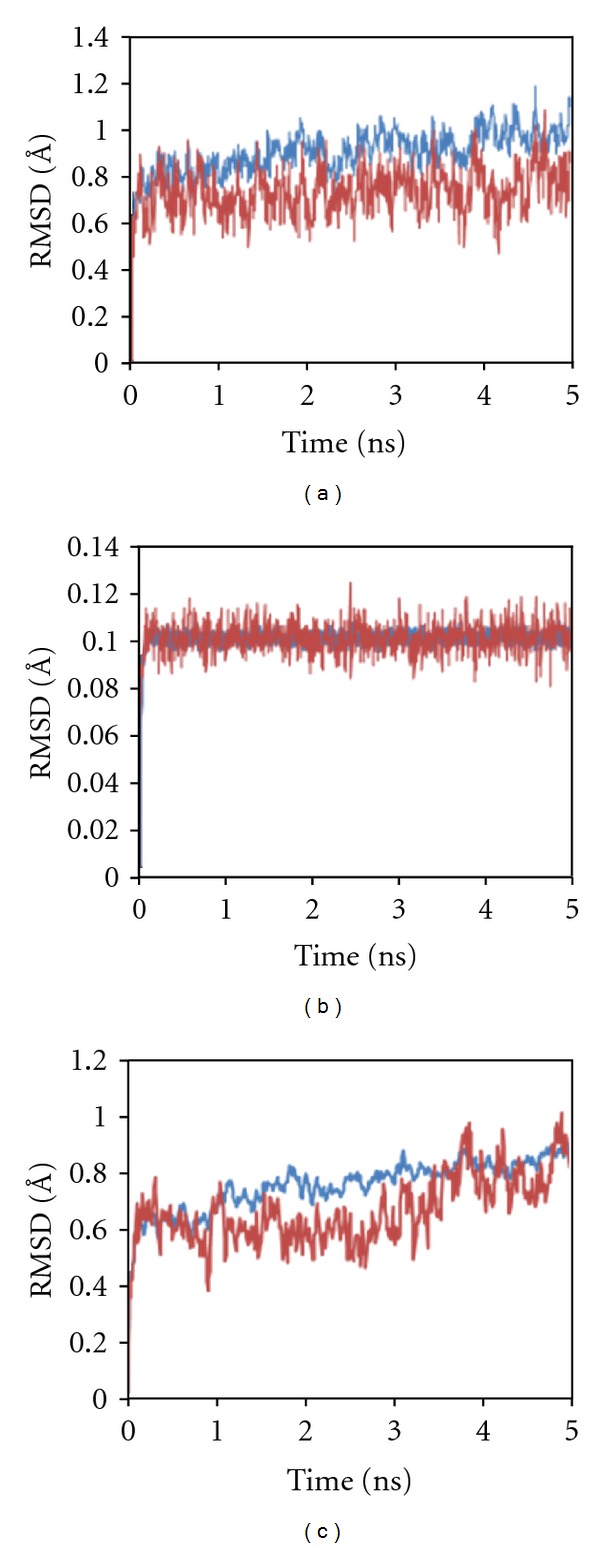
RMSD of backbone is shown for whole protein (blue) and 5 Å regions around the active site of cocrystal ligand (red). The structures, 1Z89 (a) and 3H4G (c), have lower RMSD for active site than whole protein, while 2FZ8 (b) has similar RMSD for both throughout the trajectory.

Concurrently, decreasing numbers of water molecules are observed in both the hydration shell (Figures 5(a), 6(a), and 7(a)). Although the active site of the ALR2 is highly hydrophobic, and water molecules are observed present at the opening of the active site cavity, the region included in the hydration shell. The water molecules are randomly moved away with progress of simulation while the average number of solvent water molecules remained similar to water molecules present in the crystal structures. The bridge H-bonding interaction is believed to keep these water molecules in the highly perturb site. Moreover, no severe structural changes are observed as the average backbone root mean square deviation (RMSD) is found <0.72 Å and <0.89 Å for active site residues (5 Å regions around cocrystal ligand) and whole protein respectively (Figure 8).

## 4. Conclusions

The traditional approaches for absolute free energy estimation in the MM-PB(GB)SA approach, no “nonphysical” annihilation or decoupling of the ligand in solution and bound to its receptor is necessary, nor need one simulate the partially unbound states that would be required for a potential of mean force estimate using umbrella sampling. Hence, binding free energy calculations with MM-PB(GB)SA use physical states at both end points of the binding reaction only, thereby, avoiding the need to devote computer time on intermediate states. Because the accuracy of absolute binding free energy calculations depends on a delicate balance of different energetic and entropic contributions, the calculated *in silico *binding free energy can be still considered close to the experimentally determined data. 

The water density around the ligand shows areas of high occupancy of water at these positions. They closely reproduce the position of the water molecules which were observed in the crystal structure. The life time and occupancies of water molecules also varies depending on the type of inhibitor with 3H4G having occupancy of 98.92%, 53.55% for 2FZ8, and only 16.5% for 1Z89. The occupancy ratios are also in agreement with the observed density, in which 3H4G have more density around the ligand when compared to other inhibitors. The contributions toward electrostatic free energy of solvation are very low for 3H4G as compared to other complexes. So the energy required to desolvate the binding particles in this complex is very low, while the highest energy required to desolvate is seen in 1Z89 which has a low binding energy. Moreover, binding energy pattern of the ALR2 complexes with and without water signify the importance of the water molecules in protein-ligand interaction. This study confirmed the stability of structural bridge water molecules that are always present in simulations and also revealed the presence of other structural water molecules, with lower residence time and occupancy in the complexes.

## Figures and Tables

**Table 1:** Table showing PDB ID of complexes and their inhibitors and water molecules in the active site.

PDB ID	Ligand	Ligand atom ID	Water molecule ID
1AZ1	Alrestatin	O21	HOH669
1PWL	Minalrestat	O	HOH2323
1PWM	Fidarestat	F17 N21	HOH1654 HOH1552 & 1774
1IEI	Zenarestat	O34	HOH477
1T40	IDD552	O16	HOH738
1T41	IDD552	O16	HOH5004
1Z89	Sulfonyl pyridazinone	O18	HOH1147
1Z8A	Sulfonyl pyridazinone	O18	HOH1179 & 1268
2ACQ	G6P	G6P317	HOH460
2ACU	CIT	CIT317	HOH319
2FZ8	Zopolrestat	O1	HOH1082
2FZD	Tolrestat	S1 F3	HOH355 & 389 HOH487
2PD5	Zopolrestat	O1	HOH748
2PD9	Fidarestat	N21	HOH624&816
2PDF	Zopolrestat	O1	HOH815
2PDI	Zopolrestat	O1	HOH736 & 687
2PDM	Zopolrestat	O1	HOH782
2PDW	Fidarestat	N21	HOH 750 & 615
2PDX	Zopolrestat	O1	HOH 691
2PDY	Fidarestat	N21	HOH 617
3H4G	Fidarestat	N21	HOH 2275
3G5E	Q74	N24	HOH 698

**Table 2:** IC_50_ and calculated binding free energy for ALR2 crystal structure complexes.

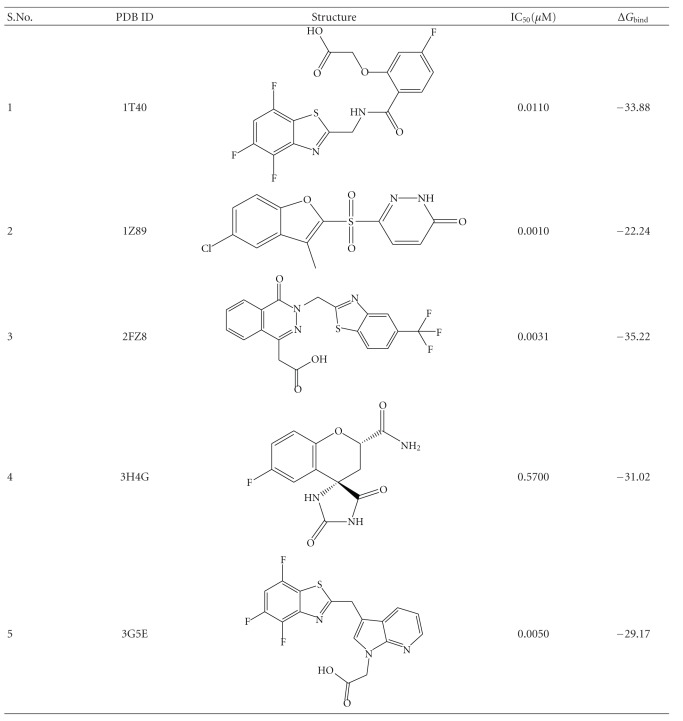

**Table 3:** Thermodynamics of binding for the 5 complexes of ALR2 calculated using MM-PBSA.

PDB ID	Δ*G*_ele_	Δ*G*_vdW_	Δ*G*_ele-int_	Δ*G*_non-polar_	Δ*G*_ele-MM_	Δ*G*_bind_^∗ ^	*G*_bind_^∗ ^
						(With water)	(Without water)
1T40	5.97	−48.31	14.96	−5.67	20.93	−25.24	−21.29
1Z89	−6.92	−44.85	36.83	−4.94	29.91	−53.71	−49.73
2FZ8	5.66	−51.96	21.49	−6.07	27.16	−18.77	−16.23
3H4G	11.81	−45.37	17.53	−5.69	29.34	−44.16	−36.60
3G5E	22.7	−46.89	4.79	−5.44	27.49	−17.55	−14.22

*Estimated by MM-GBSA program of Prime module implemented in Schrodinger *maestro* package.

**Table 4:** Hydrogen bonds data showing occupancy with distance cutoff of 5 Å along the MD simulations for 1Z89.

Donor H-X	Acceptor atom of water	1Z89	
		Occupancy (%)	Distance (Å)
62P@O18	8106@O	16.50	3.517
62P@S8	8106@O	14.50	3.753
62P@O19	8106@O	14.00	3.723
62P@N4	8106@O	11.50	3.986
62P@C3	8106@O	11.00	3.730

**Table 5:** Hydrogen bonds data showing occupancy with a distance cutoff of 5 Å for 2FZ8 during the MD simulations.

Donor H-X	Acceptor atom of Water	2FZ8	
		Occupancy (%)	Distance (Å)
ZST@C9	328@O	53.55	3.730
ZST@N3	328@O	49.73	4.378
ZST@C10	328@O	46.45	4.435
ZST@N1	328@O	36.09	4.614
ZST@O1	328@O	15.00	4.747

**Table 6:** Hydrogen bonds data showing occupancy with a distance cutoff of 5 Å in the MD simulations for 3H4G.

Donor H-X	Acceptor atom of water	3H4G	
		Occupancy (%)	Distance (Å)
FID@C9	346@O	98.82	3.683
FID@O10	346@O	97.55	3.765
FID@C11	346@O	95.27	4.047
FID@C19	346@O	91.91	4.299
FID@O6I	346@O	89.64	3.423
